# The Role of Digit- and Pacifier-Sucking Habits on Malocclusion Development in Children: Anterior Open Bite and Posterior Crossbite—A Systematic Review & Meta-Analysis

**DOI:** 10.3390/dj14010055

**Published:** 2026-01-14

**Authors:** Arvin Faryad, Susana Muwaquet Rodriguez, Tawfiq Hijazi Alsadi

**Affiliations:** 1 Faculty of Medicine and Health Science, Catholic University of Valencia (UCV), C/Quevedo, 2, 46001 Valencia, Spain; arfar@mail.ucv.es; 2 Department of Restorative Dentistry and Endodontics, Faculty of Medicine and Health Science, Catholic University of Valencia (UCV), C/Quevedo, 2, 46001 Valencia, Spain; 3 Department of Orthodontics, Faculty of Medicine and Health Science, Catholic University of Valencia (UCV), C/Quevedo, 2, 46001 Valencia, Spain

**Keywords:** anterior open bite, digit sucking, malocclusion, non-nutritive sucking, pacifier sucking, posterior crossbite, thumb sucking

## Abstract

**Background/Objectives:** Malocclusion is one of the most prevalent oral health concerns in paediatric dentistry, with anterior open bite (AOB) and posterior crossbite (PCB) being among the most common forms. Non-nutritive sucking habits (NNSHs), including digit-sucking habits (DSHs) and pacifier-sucking habits (PSHs), have been linked to malocclusion development. While both habits are known to impact dental and skeletal development, their comparative effects remain unclear. This systematic review and meta-analysis aims to determine the difference in the development and prevalence of anterior open bite and posterior crossbite between patients with digit-sucking and pacifier-sucking habits. **Materials and Methods:** An exhaustive review of the literature was conducted on the 25 November 2024 across three databases, namely EBSCOhost (including PubMed-Medline), Web of Science and Scopus. The following PICO question was constructed for the systematic review: “In children and teenagers, is there a difference in the development and prevalence of malocclusions (Anterior Open bite & Posterior Crossbite) between patients with a history of digit sucking habits and patients with a history of pacifier sucking habits?”A meta-analysis was also performed with the selected studies, and the software used to carry out the meta-analysis was R 4.3.1 (R Core Team (2023)). **Results:** From the initial search, 102 articles were found and a further 11 articles were obtained from manual findings. 12 articles were included in the final systematic review and meta-analysis. The meta-analysis indicated that the risk of AOB and PCB was increased by both DSH and PSH. **Conclusions:** Both DSH and PSH significantly increased the risk of AOB and PCB. PSH posed a significantly higher risk than DSH for PCB development (OR = 2.66, *p* < 0.001), while no significant difference in AOB prevalence was observed between DSH and PSH (OR = 1.77, *p* = 0.150).

## 1. Introduction

Within pediatric dentistry, malocclusion is a significant concern, particularly when specific habits are present in children, such as digit-sucking habits (DSHs) or pacifier-sucking habits (PSHs), which can lead to orthodontic problems and require prompt intervention. The primary dentition serves as the foundation for permanent teeth, playing a crucial role in maintaining space and guiding proper occlusion for future tooth development [1]. The concept of “normal occlusion” was described by John Hunter Carabelli in the 18th century, referring to the relationship of the cusps and intercuspation of the maxillary and mandibular teeth [2]. Malocclusion is defined as a developmental disorder resulting from genetic and environmental factors affecting the jaws, tongue, and facial muscles of the craniofacial complex [3]. Specifically, malocclusion describes any deflection from the normal alignment of teeth relative to other teeth in the same arch or to teeth in the opposing arch [4]. It is the third-most prevalent oral disease after dental caries and periodontal disease [5], thereby making malocclusion a public health problem due to its prevalence and negative consequences, including compromised quality of life, social interaction difficulties, and psychological impacts [5]. Thus, early identification, intervention, and prevention are crucial.

**Figure 1:**
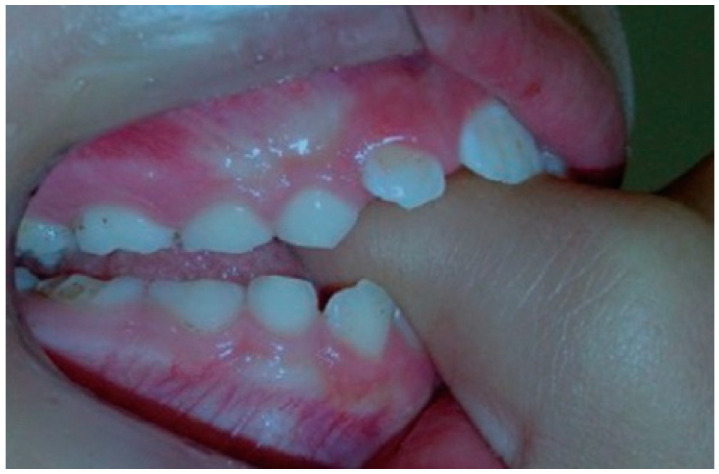
Interposition of thumb in mouth.

**Figure 2:**
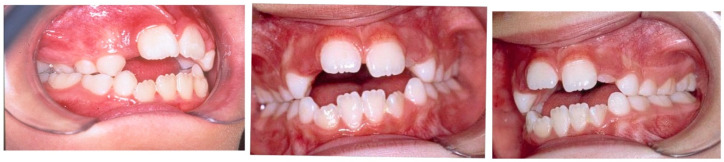
Clinical case of AOB caused by digit-sucking.

**Figure 3:**
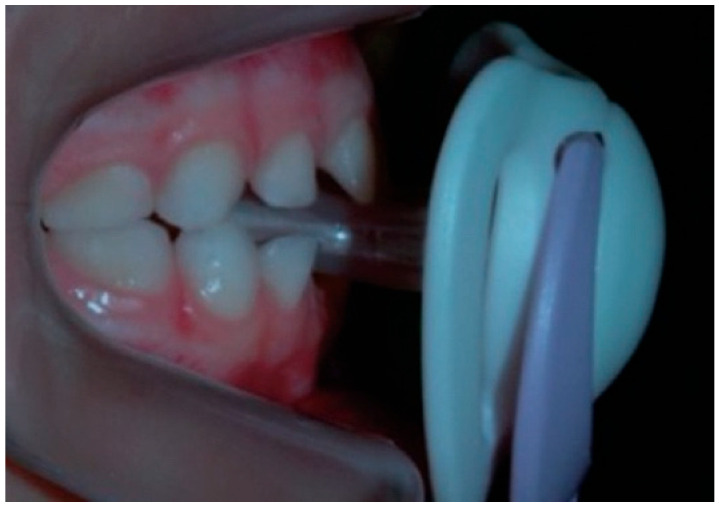
Interposition of pacifier in mouth.

**Figure 4:**
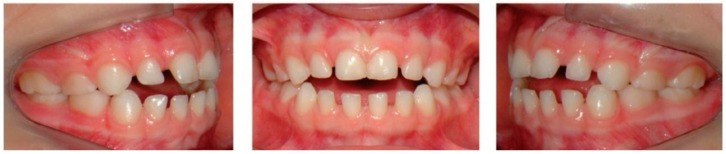
Clinical case of AOB caused by pacifier-sucking.

Malocclusions may appear in both primary and permanent dentitions, with anterior open bite (AOB) and posterior crossbite (PCB) being among the most common [6]. AOB is characterized by the absence of vertical overlap between incisors when posterior teeth are in occlusion [7]. It can arise from skeletal or dental origins and typically causes esthetic and functional concerns, such as difficulty biting food and impaired speech [8]. A key etiological factor for AOB is digit-sucking (as seen in Figure 1). Clinical features of dental AOB include proclination of anterior teeth, spacing, and a narrow maxillary arch (as seen in Figure 2). Skeletal AOB often involves excessive vertical growth, increased lower anterior facial height, decreased upper facial height, and a narrow maxillary arch [8]. Etiological factors for AOB include digit-sucking (as seen in Figure 1), pacifier use (as seen in Figure 3), abnormal tongue thrusting, and mouth breathing, emphasizing the importance of early habit correction to prevent more complex future interventions [9]. AOB caused by pacifier sucking can be seen in the clinical case (as seen in Figure 4). Treatment approaches include habit-breaking appliances, orthodontics for dental AOB, and potentially surgical intervention for severe skeletal AOB after growth cessation [10].

Posterior crossbite is defined by an abnormal transverse relationship between the upper and lower dental arches, often resulting from a narrowed maxillary arch [11]. PCB may be unilateral or bilateral and categorized as dental, functional, or skeletal. It is highly prevalent in primary dentition and is among the common orthodontic problems in early occlusal development [12]. Etiological factors of PCB include hereditary factors, such as maxillary hypoplasia or mandibular hyperplasia, as well as environmental factors like non-nutritive sucking habits [13]. Diagnosis involves clinical and radiographic examinations, with treatments including occlusal adjustments, orthodontic appliances, or orthodontic brackets, depending on the dentition stage (primary, mixed, or permanent) [14].

Non-nutritive sucking habits (NNSHs) are prevalent in children, providing emotional comfort but potentially causing dental issues if prolonged beyond the early years. Both digit-sucking and pacifier-sucking exert biomechanical forces on dental arches, disrupting normal growth and alignment, thus significantly increasing the risk for developing AOB and PCB [15,16]. Despite the soothing role of these habits, prolonged digit or pacifier use beyond the age of 3–4 years increases risks of persistent malocclusions [17]. Studies suggest that digit-sucking may lead to a higher prevalence of AOB, whereas pacifier-sucking has a more pronounced impact on PCB [18]. Considering these differential impacts, further systematic evaluation is necessary to inform pediatric dentists and parents regarding the comparative consequences of these habits, emphasizing timely education and appropriate intervention strategies, such as habit-breaking appliances [19].

### 1.1. Hypothesis

There is a significant difference in the prevalence of anterior open bite between children with digit-sucking habits and those with pacifier-sucking habits. Additonally, there is a significant difference in the prevalence of posterior crossbite between children with digit-sucking habits and those with pacifier-sucking habits.

### 1.2. Objectives

***General objective:***

To know the difference in the prevalence of malocclusion development in the vertical and transverse plane (anterior open bite and posterior crossbite) between children and teenagers with a history of digit-sucking habits and pacifier-sucking habits.

***Specific objectives:***

I. To determine the prevalence of anterior open bite in children with digit-sucking habits vs. non-digit-sucking habits and pacifier-sucking habits vs. non-pacifier-sucking habits.
II. To determine the prevalence of posterior crossbite in children with digit-sucking habits vs. non-digit-sucking habits and pacifier-sucking habits vs. non-pacifier-sucking habits.
III. Through a meta-analysis, to analyze the overall association between digit- and pacifier-sucking habits in the development of anterior open bite and posterior crossbite, and the clinical implications

## 2. Materials and Methods

This systematic review rigorously followed the PRISMA 2020 guidelines (*Preferred Reporting Items for Systematic Reviews and Meta-Analyses*) (Table S1). The following structure was used to formulate the review question:

- P (Population): children and teenagers.
- I (Intervention): history of digit-sucking habits; history of pacifier-sucking habits.
- C (Comparison): patients without any non-nutritive sucking habits.
- O (Outcome): development and prevalence of malocclusions (anterior open bite and posterior crossbite).

The PICO question was as follows: “*In children and teenagers, is there a difference in the development and prevalence of malocclusions (anterior open bite and posterior crossbite) between patients with a history of digit-sucking habits and patients with a history of pacifier-sucking habits?*”

### 2.1. Eligibility Criteria

The **inclusion** criteria for the studies included had to adhere to the following:

- **Type of study:** Publications in the English language; published from January 2000 to December 2024; observational studies, including case–control studies, cohort studies, cross-sectional studies, and longitudinal studies; experimental studies, including randomized controlled trials (RCTs) and non-randomized studies.
- **Type of patient/teeth:** Children and teenagers (both genders); deciduous dentition, mixed dentition, or permanent dentition; history of pacifier-sucking habit or history of digit-sucking habit; normal number, size, and shape of all teeth in the dentition.
- **Type of intervention:** Observational exposure to pacifier-sucking habits or digit-sucking habits (e.g., frequency, duration, or history of habit presence); history of pacifier-sucking habit or digit-sucking habit/PSH or DSH present at the time of the study.
- **Type of outcome variables:** Studies reporting measurable malocclusion outcomes (prevalence of anterior open bite and posterior crossbite); studies providing quantitative data for statistical analysis (e.g., prevalence rates, relative risk, and *p*-values).

The **exclusion** criteria for the studies included had to adhere to the following:

- **Type of study:** Case reports or case studies with insufficient sample sizes for generalization (less than 10); animal or in vitro studies; systematic reviews and meta-analyzes.
- **Type of patient/teeth:** Major tooth destruction (due to caries or trauma) or reconstruction; systemic diseases and/or neurological diseases affecting craniofacial growth (e.g., cleft palate); previous orthodontic treatment.
- **Type of intervention:** Nutritive sucking habits (e.g., breastfeeding and bottle-feeding) or interventions unrelated to non-nutritive sucking habits.
- **Type of outcome variables:** Studies lacking specific malocclusion-related outcomes (e.g., general oral health or unrelated dental conditions); studies with insufficient or non-quantitative data that cannot be included in a meta-analysis; studies without any data on the specific malocclusions of interest (studies without any data on posterior crossbite and anterior open bite).

### 2.2. Search Strategy and Study Selection

From the derived PICO question, the search strategy was developed. Subsequently, Boolean operators (AND, OR) were used alongside selected specific keywords. The initial keywords used were “non-nutritive sucking”, “digit sucking”, “thumb sucking”, “pacifier sucking”, “malocclusion”, “anterior open bite”, and “posterior cross bite”. These keywords were then combined with Boolean operators, and individual searches were made across each database to obtain the most relevant studies addressing the PICO question. Filters applied included articles published from 2000 to 2024 and publications in the English language.

The search across all three databases was as follows:

EBSCOhost: (non nutritive sucking) AND (digit sucking) OR (thumb sucking) AND (pacifier sucking) AND (malocclusion) AND (anterior open bite) AND (posterior cross bite).

Web of Science: (non nutritive sucking) AND (digit sucking) OR (thumb sucking) AND (pacifier sucking) AND (malocclusion) AND (anterior open bite) AND (posterior cross bite).

Scopus: “Non nutritive sucking” AND “thumb sucking” AND “pacifier sucking” AND “malocclusion” AND “posterior crossbite” OR “anterior open bite”.

### 2.3. Selection Process of the Studies

**Figure 5:**
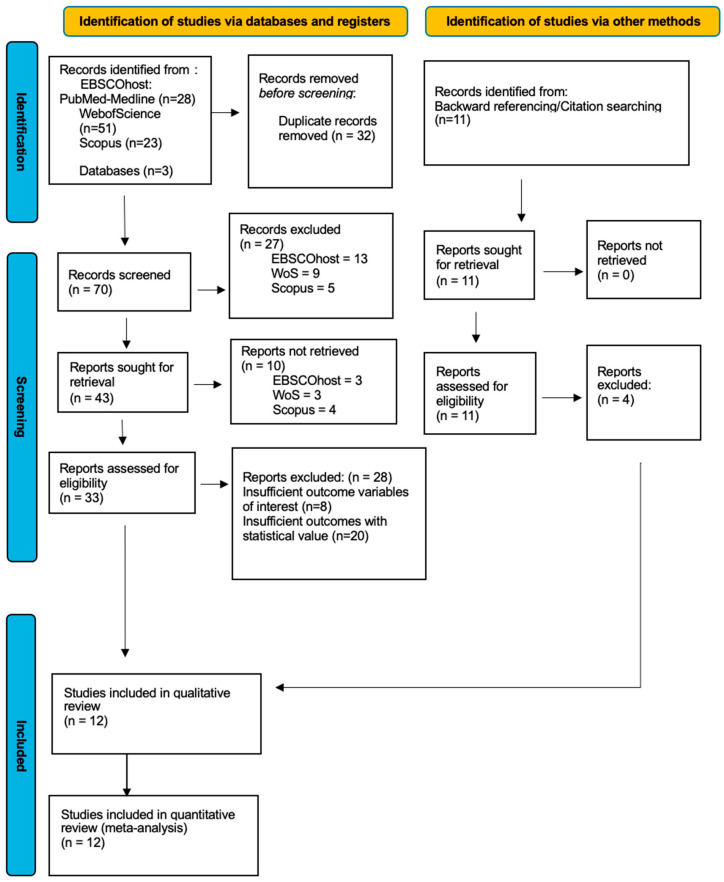
PRISMA flowchart demonstrating the scheme that was followed in the selection of articles.

Study selection was carried out in three stages by two reviewers (A.F and T.H.A). Following the advanced search using the specific date criteria, keywords, and English language restriction, the articles/studies that were found went through an initial screening procedure. Before screening any studies, the first stage of this selection process involved pre-screening duplicate removal, meaning that any duplicates found across databases were removed. After the removal of all duplicates, a total of 70 studies remained and underwent preliminary screening involving two parts. Firstly, the titles of these articles were read, and any of that were deemed as irrelevant to the topic or did not meet the inclusion criteria were removed. Secondly, the abstracts of the articles were screened, which involved a brief assessment and overview of the introduction, methods, results, and conclusion. From the abstract screening, any further articles were removed if they were considered not relevant to the topic or failed to answer the PICO question. After completion of the preliminary screening, a total of 33 articles remained across the three databases and were subjected to a more rigorous screening, which involved a complete assessment of the full texts, and relevant data from the selected studies were obtained. After this stage, any remaining studies that could not be retrieved for given reasons were excluded from the final systematic review. The remaining articles were included in the systematic review and meta-analysis. Disagreements between reviewers regarding inclusion/exclusion of articles were resolved through discussion. Inter-examiner agreement regarding final inclusion/exclusion of articles was obtained using Cohen’s kappa test, following the scale proposed by Landis and Koch [20]. Inter-examiner agreement was considered almost perfect (k = 0.87). This represents the final stage of the selection screening, and this selection process is demonstrated by the constructed PRISMA flowchart seen in Figure 5, with a final inclusion of 12 articles.

### 2.4. Data Extraction

A table was constructed to present the relevant information and data extracted from the studies. It included information regarding the title and authors of the articles, year of publication, type of study conducted, sample size, how the malocclusion was measured (if stated), how the habit was identified (if stated), and lastly, the duration of the habit/s (if stated).

To enhance validity and reliability and to avoid heterogeneity in the extracted results in terms of the variables used, the presence and absence of AOB and PCB were selected as two consistent outcome variables to be analyzed as a reflection of the presence/absence of DSH and PSH across the studies in the meta-analysis.

Other statistics, such as unilateral PCB or AOB > 3 mm, which are considered valid outcome variables in the evaluation of DSH and PSH, were excluded from the meta-analysis due to a lack of statistical data and, additionally, to ensure homogeneity in the reported results by keeping outcome variables as similar as possible. Due to insufficient statistical data with regard to teenagers presenting with these habits, teenagers were excluded from the meta-analysis, as all the studies provided information solely on children and infants.

For the meta-analysis, the data extracted from the studies included the total number of participants in each study, the number of participants present with either DSH or PSH who presented with AOB or PCB, the number of participants without either DSH or PSH who presented with AOB or PCB, the number of participants present with either DSH or PSH who presented without AOB or PCB, and, finally, the number of participants without either DSH or PSH who presented without AOB or PCB.

### 2.5. Data Synthesis

A meta-analysis was carried out to evaluate the extracted data in order to compare the prevalence/risk of AOB and PCB between patients with DSH and PSH. The software used was R 4.3.1 (R Core Team (2023). R: A language and environment for statistical computing. R Foundation for Statistical Computing, Vienna, Austria. URL http://www.R-project.org/, accessed on 15 January 2025). Researchers have conducted an exhaustive systematic review of the available literature, arriving at a final inclusion of 12 studies. The primary outcomes of the study were the prevalence rates of AOB and PCB.

It should be kept in mind that the patient population in each study had different degrees of AOB and PCB, which in principle is already a source of heterogeneity (it is likely that more severe cases of AOB and PCB were patients present with more intense frequency/duration of DSH/PSH). This limitation could not be solved, as the majority of the included studies did not evaluate the frequency/duration of the habits, nor did they evaluate the severity of AOB or PCB.

Most of the studies (10/12) compare prevalence of anterior open bite (AOB) between children with and without digit-sucking habits (DSHs). Additionally, nine of the included studies compared the prevalence of anterior open bite (AOB) between children with and without pacifier-sucking habits (PSHs).

Regarding the prevalence of posterior crossbite (PCB), ten of the included studies compared both DSH and non-DSH groups. In addition, ten studies compared PCB rates between PSH and non-PSH groups.

The following table (Table 1) summarizes the number of patients examined in each study (AOB/PCB) per group (DSH, non-DSH, PSH, and non-PSH).

The following meta-analysis will be conducted in the AOB study:

1. A random-effects model will be used to estimate the overall effect measure (AOB rate) for each group (DSH/control). A restricted maximum likelihood estimator of heterogeneity will be used. Forest graphs will be plotted in order to visualize results with 95% confidence intervals. Regarding heterogeneity analysis, Cochran’s Q test was applied, and the I^2^ index of heterogeneity was also calculated, representing the proportion of between-study variability compared to total variability. Funnel graphs were also generated to explore potential publication bias, and Egger’s test was carried out to measure the impact of this type of bias (despite its low power in small samples). Wilson’s correction was used to estimate heterogeneity estimators under absolute individual rates (0% and 100%).
2. ***Comparison between DSH and control group***. As the effect measure, the odds ratio (OR) will be calculated (expressed as log OR because of symmetric and normality characteristics) from a meta-analysis with a random-effects model. Corresponding Z statistics, *p*-values, and 95% confidence intervals were obtained, and these results were represented using forest plots.

Both meta-analysis 1 and 2 will be repeated to assess the ***effect of PSH***.

3. In order ***to compare the effect of DSH* vs. *PSH on the risk of AOB***, a model for the weighted mean difference (WMD) of previously calculated effect measures (log OR) will be conducted.

All the previous methodology will be repeated for statistical purposes in the second study (**PCB**). The *level of significance* that was used in the analysis was 5% (α = 0.05).

The software used was R 4.3.1 (R Core Team (2023). R: A language and environment for statistical computing. R Foundation for Statistical Computing, Vienna, Austria. Available online: http://www.R-project.org/ (accessed on 15 January 2025)

## 3. Results

From the initial search, a total of 102 articles were obtained from the following databases: Web of Science (*n* = 51), EBSCO (*n* = 28), and Scopus (*n* = 23). A further 11 articles were obtained through manual searching. Following this, a total of 32 duplicate records were manually removed prior to screening. Next, a preliminary screening was conducted by reviewing the article titles, leading to the removal of 27 articles that did not meet the inclusion criteria or were irrelevant to the topic. Following this step, abstracts from the remaining 43 articles were retrieved and reviewed. After assessing the abstracts, 10 more articles were excluded because they were deemed irrelevant or did not address the PICO question. Finally, an in-depth evaluation was performed on the remaining 33 articles through a detailed review of the full texts. Articles found unsuitable for inclusion were excluded, with explicit reasons provided for their removal.

The reasons for the exclusion of these articles were due to either insufficient outcome variables of interest (*n* = 8) or insufficient outcomes with statistical value to perform a meta-analysis (*n* = 20). The selection process of the studies therefore resulted in a total of 12 articles that were eligible following screening.

The primary aim of the research was to assess and compare the effects of DSH vs. PSH on the risk of AOB and PCB development. The review and combination of results from several studies were conducted using meta-analysis. The researcher looks to integrate the information derived from the different reports to reach a general conclusion.

### 3.1. Comparison of DSH vs. PSH Effects on AOB

Freire et al. [21] reported strong differences in AOB rate between DSH and non-DSH (71.4% vs. 23.3%, log OR = 2.11) groups. However, differences in AOB rate between PSH and non-PSH groups were weaker and showed an inverse tendency (25.1% vs. 27.9%; log OR = −0.14). Therefore, for this study, a stronger effect of DSH than PSH on AOB development was estimated.

The following table (Table 2) includes estimators of both PSH and DSH effect measures previously estimated by models:

The following meta-analysis estimated a mean difference:

$$\log{OR}_{PSH}-\log{OR}_{DSH}=\log\frac{{OR}_{PSH}}{{OR}_{DSH}}$$

That is to say, the mean differences is equivalent to the ratio of ORs expressed on the logarithmic scale.

**Figure 6:**
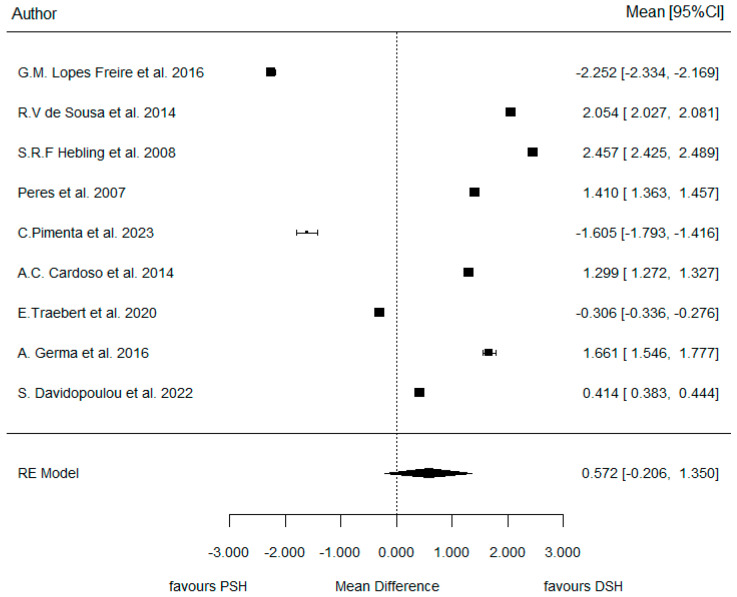
Forest plot comparing PSH and DSH groups for AOB prevalence [3,21,22,23,24,25,26,27,28].

The meta-analysis concluded a WMD = 0.57 (representing the difference between both log Ors of PSH-DSH) (as seen in the Forest plot of Figure 6). In other words, the ratio of ORs (PSH/DSH) was 1.77, indicating that the risk of AOB increased by +77% when a pacifier was used compared with digit-sucking.

However, this effect did not reach statistical significance (*p* = 0.150) (as seen in Table 3). Therefore, we will accept the null hypothesis of similar consequences, indicating no significant difference in the development and prevalence of AOB between DSH and PSH.

**Figure 7:**
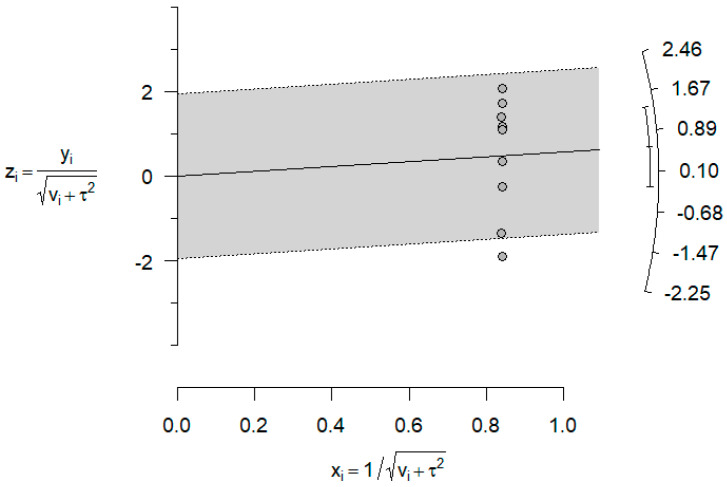
Galbraith plot comparing DSH vs. PSH groups for AOB rate.

However, the heterogeneity associated with the model was very high (I^2^ = 99.9%). The following Galbraith graph (Figure 7) shows that Freire et al. [21] was the most heterogeneous article compared with the rest.

**Figure 8:**
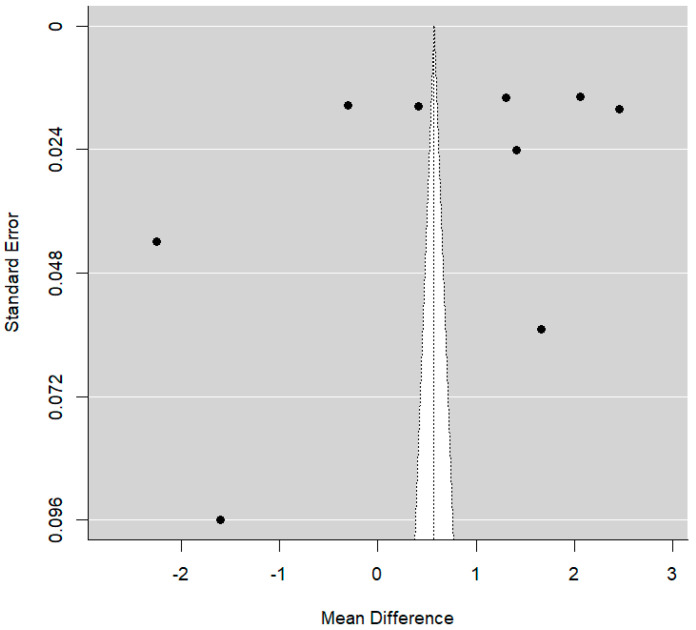
Funnel plot comparing DSH vs. PSH groups for AOB rate.

It was noted that some articles involving higher standard errors usually reported more favorable clinical examinations for PSH (*p* = 0.033), as seen in Figure 8 below.

### 3.2. Comparison of DSH vs. PSH Effects on PCB

Freire et al. [21] reported differences in PCB rate between DSH and non-DSH groups (0% vs. 13.1%, log OR = −1.49). However, differences in PCB rate between PSH and non-PSH groups showed an inverse tendency (14.6% vs. 4.9%; log OR = 1.19). Therefore, for this study, a stronger effect of PSH than DSH was estimated.

The following table (Table 4) includes estimators of both PSH and DSH effect measures previously estimated by models:

The following meta-analysis estimated a mean difference:

$$\log{OR}_{PSH}-\log{OR}_{DSH}=\log\frac{{OR}_{PSH}}{{OR}_{DSH}}$$

That is to say, the mean differences is equivalent to the ratio of ORs expressed on the logarithmic scale.

**Figure 9:**
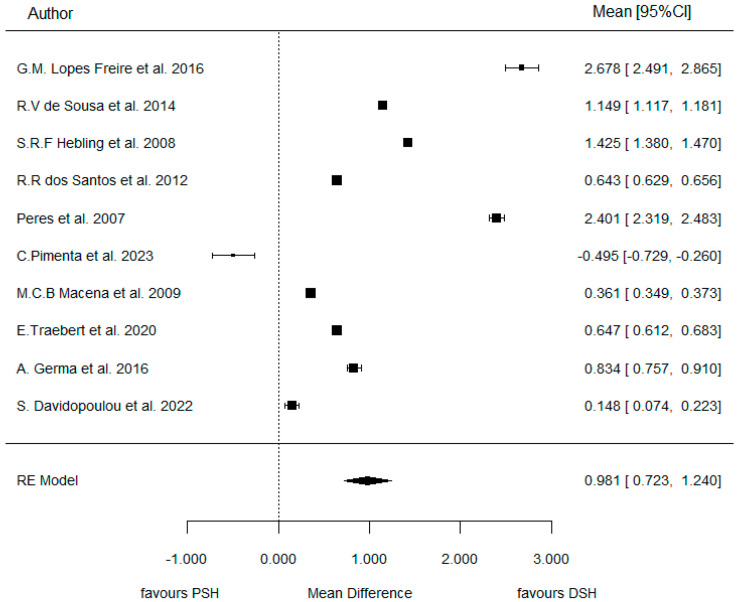
Forest plot comparing PSH and DSH groups for PCB prevalence [3,21,22,23,24,25,26,28,29,30].

The meta-analysis concluded a WMD = 0.98 (as seen in the Forest plot in Figure 9) (representing the difference between both log ORs of PSH-DSH). In other words, the ratio of ORs (PSH/DSH) was 2.66, indicating that the risk of PCB increased by +166% when pacifier-sucking was present compared to digit-sucking.

This effect reached statistical significance (*p* < 0.001); therefore, we will reject the null hypothesis of similar consequences.

**Figure 10:**
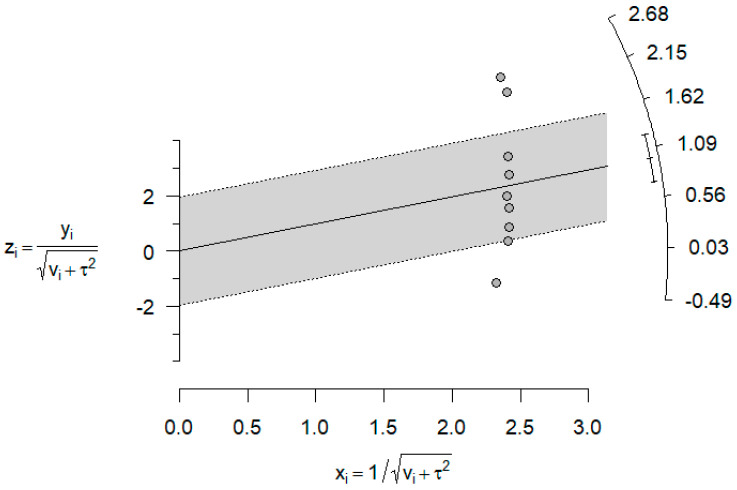
Galbraith plot comparing DSH vs. PSH groups for PCB rate.

**Figure 11:**
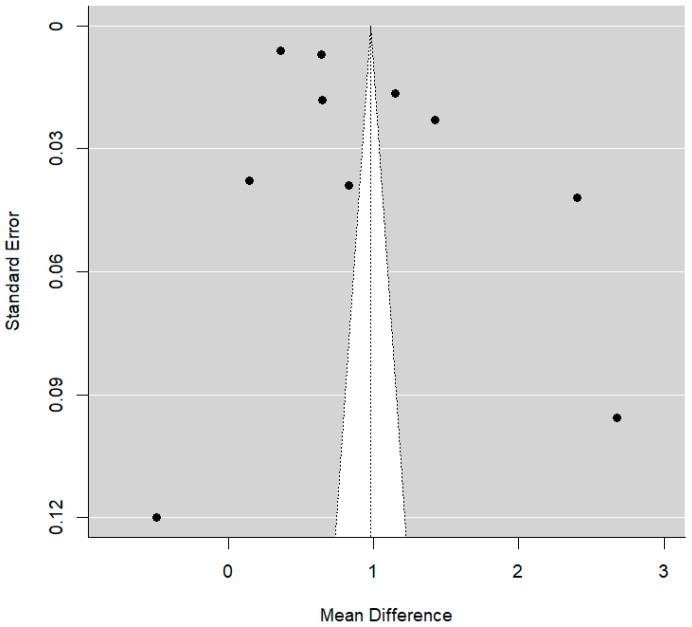
Funnel plot comparing DSH vs. PSH groups for PCB rate.

We can observe from Table 5 that the heterogeneity associated with the model is very high (I^2^ = 99.9%). The following Galbraith graph (Figure 10) shows that Freire et al. [21], Peres et al. [3], and Pimenta et al. [22] showed especially large measures of the effect:

With regard to publication bias, there are no signs of publication bias, as demonstrated in the funnel plot below (Figure 11).

## 4. Discussion

This systematic review and meta-analysis aimed to determine the difference between the role of digit-sucking habits (DSHs) and pacifier-sucking habits (PSHs) on the development and prevalence of anterior open bite (AOB) and posterior crossbite (PCB). Twelve studies were analyzed to evaluate whether PSH and DSH had differential influences on vertical and transverse malocclusions.

All included studies concluded that non-nutritive sucking habits (NNSHs) significantly contributed to malocclusion development, with both DSH and PSH significantly associated with increased prevalence of AOB and PCB. The meta-analysis indicated that both habits had similar impacts on AOB, whereas PSH showed a stronger association with PCB.

### 4.1. General Objective

The general objective was to determine the difference in malocclusion prevalence between children with histories of DSH versus PSH. The results demonstrated both habits are risk factors for malocclusion, significantly increasing the prevalence of AOB and PCB. Freire et al. [21] noted that children with NNSH had a 2.55-times greater risk of malocclusion. They found that PSH had significantly greater effects in the transverse plane (*p* = 0.013), while DSH showed greater effects in the vertical plane (*p* = 0.001). Tanny et al. [31] supported this by noting that thumb sucking was critical for AOB formation. Additionally, pacifier use likely increases PCB prevalence due to altered muscle dynamics and tongue positioning, as highlighted by Sousa et al. [23], who observed higher AOB and PCB prevalence with pacifier use beyond 36 months. Sousa et al. also found that advancing age correlated with reduced AOB prevalence if habits ceased.

Contrarily, R.V. de Sousa et al. [23] found no significant association between DSH and PCB, suggesting that other factors, like genetics and respiratory patterns, may influence transverse malocclusions. Germa et al. [24] also identified preterm birth as another potential risk factor for early PCB alongside NNSH.

### 4.2. Specific Objectives

The specific objectives involved comparing AOB and PCB prevalence for DSH vs. non-DSH and PSH vs. non-PSH, and analyzing the associations between these habits.

### 4.3. AOB Prevalence: DSH vs. Non-DSH and PSH vs. Non-PSH

For the non-DSH group, the meta-analysis found a mean AOB rate of 15.8%, with high heterogeneity (I^2^ = 98.6%). This variability may reflect unrecorded factors like prolonged bottle-feeding or incomplete questionnaires, particularly in Peres et al. [3], where the mean AOB rate reached 40.9%. The DSH group had a notably higher mean rate (42.6%), confirming that DSH significantly increased AOB risk (OR = 5; *p* < 0.001), aligning with findings by Pimenta et al. [22] (*p* = 0.02) and Tanny et al. [31], where AOB prevalence reached 78.7% among digit suckers.

For PSH, the mean AOB prevalence was 47.8% compared to 9.1% in the non-PSH group (OR = 7.39; *p* < 0.001), highlighting PSH as a significant risk factor. Studies by Hebling et al. [25], Santos et al. [29], and Traebert et al. [26] supported these findings, confirming that pacifier use significantly increased AOB prevalence, particularly beyond the age of three years. Cardoso et al. [27] also showed that PSH beyond three years significantly increased AOB risk (OR = 44; *p* < 0.001).

### 4.4. PCB Prevalence: DSH vs. Non-DSH and PSH vs. Non-PSH

DSH had a higher, but statistically insignificant, PCB risk (OR = 1.27; *p* = 0.055), with mean PCB rates nearly identical (11.9% vs. 11.6% non-DSH). Sousa et al. [23] supported this insignificant association, suggesting that DSH slightly increased PCB risk but lacked strong evidence. Davidopoulou et al. [28] similarly found limited PCB prevalence with digit-sucking.

In contrast, PSH showed a significantly higher PCB prevalence (23.0%) versus non-PSH (6.9%), substantially increasing PCB risk (OR = 2.80; *p* < 0.001). Santos et al. [29] confirmed this association (*p* < 0.0001), and Freire et al. [21] also supported the significant impact of PSH on transverse malocclusion development.

### 4.5. Overall Associations: DSH vs. PSH

Overall, the meta-analysis indicated that DSH increased the odds of AOB fivefold (OR = 5.0), while PSH increased the odds more than sevenfold (OR = 7.39), although direct comparisons between habits for AOB yielded no statistically significant difference (OR = 1.77; *p* = 0.150). The analysis had high heterogeneity (I^2^ = 99.9%), particularly due to Freire et al. [21].

For PCB, PSH significantly increased the odds compared with DSH (OR = 2.66; *p* < 0.001). Macena et al. [30] and Sousa et al. [23] confirmed that prolonged pacifier use strongly influenced PCB. Peres et al. [3] also suggested that bottle-feeding may contribute similarly due to mechanical factors on dental alignment.

The findings highlight a clear need for early intervention and parental education to reduce malocclusion risk from prolonged sucking habits. Public health approaches should prioritize preventive strategies, including encouraging breastfeeding and eliminating harmful habits by the age of three to four years. Healthcare professionals must educate families about the potential consequences if such habits persist beyond the recommended ages [25,29].

### 4.6. Limitations

Despite the strengths of carrying out a meta-analysis, several limitations of this study should be acknowledged. High heterogeneity was observed in the majority of the meta-analyses, which may reflect differences in study design, population characteristics, measurement methods, and diagnostic criteria, making it somewhat difficult to draw conclusions without an obvious source of variation that challenges it. This study aimed to evaluate solely patients either with a history of PSH/DSH or with current and ongoing PSH/DSH; therefore, other factors, such as habit duration, frequency, and intensity, were not taken into consideration. Differences in habit duration and participant age were not consistently reported across the included studies. For example, prolonged pacifier-sucking beyond three years of age had been linked to more severe malocclusion development, but not all studies accounted for habit duration. A subgroup analysis based on these factors could potentially have aided in minimizing the heterogeneity; therefore, this resulted in a limitation of the study. However, data with regard to habit duration and intensity were limited, which restricted the subgroup analysis from being performed; therefore, more data and future research with regard to factors affecting these habits should be conducted. It is also important to note additional factors that could have played a role in the contribution to AOB and PCB, such as poor posture, postural asymmetry at birth that persist into later life, and, most importantly, speech disorders, which can also be potential causes. Therefore, other factors should also be considered in further research studies. Future research should also aim in standardizing the diagnostic criteria for AOB and PCB and assessing the long-term effects of early intervention. In hindsight, heterogeneity could potentially have been avoided if AOB/PCB has been classified with measurements in mm, thereby focusing only on a specific diagnostic criterion. However, due to insufficient data, the meta-analysis relied on the presence/absence of AOB/PCB. While this approach identifying the prevalence of malocclusions associated with the habit, it lacks insight into the degree and severity of malocclusions, which could have minimized heterogeneity.

Another limitation is associated with the fact that pacifier use and digit-sucking are confounder variables, because it cannot be determined whether malocclusion resulted from bottle-feeding or pacifier/thumb sucking. Memory bias is also a significant limitation, as memory bias may have occurred regarding the history of sucking habits. Additionally, upon reviewing the quality of the studies, as assessed using the Newcastle–Ottawa Scale (Tables S2 and S3), the studies by Freire et al. 2016 [21], Pimenta et al. 2023 [22], and Macena et al. 2009 [30] were rated as six stars out of ten and were therefore deemed “satisfactory” in terms of quality and therefore were not given a “good quality” or “very good quality” rating. This indicates potential biases in the sample selection and measurement consistency.

Finally, it must be acknowledged that a limited number of studies were included in this systematic review and meta-analysis (*n* = 12), despite performing an exhaustive review of the studies available. Therefore, further clinical studies are required on the use of pacifiers and digit-sucking on malocclusion development in the vertical and transverse planes. Most of the studies were cross-sectional, and despite the multiple benefits of cross-sectional studies, such as being quick, cost-effective, and allowing easy data collection, cross-sectional studies provide data at only one point in time and thus lack data on changes over time. Therefore, a limitation of these studies is that it is very difficult to establish cause-and-effect relationships or determine whether the exposure preceded the outcome, and that these studies may not be representative of the broader population. Longitudinal studies are therefore needed to assess long-term effects and cause-and-effect relationships. Before performing this study, it was desired to evaluate teenagers in the permanent dentition as well. This is because teenagers may previously have had the habit and subsequently stopped the habit; therefore, evaluation in the permanent dentition would provide stronger insight into the risks of the habit. Nevertheless, the cross-sectional studies did help to identify the risks of habits associated with the prevalence of malocclusions; however, longitudinal studies would better evaluate long-term risks and consequences, further demostrating that future studies are still required.

## 5. Conclusions

- The information and results integrated from a variety of different studies provided sufficient evidence to conclude the following:
  - There is no significant difference in the prevalence of AOB between children with digit- or pacifier-sucking habits.
  - There is a significant difference in the prevalence of PCB between children with digit- or pacifier-sucking habits, with the prevalence of PCB associated with PSH being significantly higher.
- The meta-analysis provided significant data to conclude the following:
  - The risk of AOB for DSH was significantly higher than for non-DSH (OR = 5; *p* < 0.001).
  - The risk of AOB for PSH was significantly higher than for non-PSH (OR = 7.4; *p* < 0.001).
  - The risk of PCB was not significantly higher for DSH compared with non-DSH (OR = 1.27; *p* = 0.055).
  - The risk of PCB was significantly higher for PSH compared with non-PSH (OR = 2.80; *p* < 0.001).

## Figures and Tables

**Table 1:** Number of patients examined for AOB and PCB studies.

	AOB Study	PCB
**DSH**	557	943
**Non-DSH**	4453	6193
**PSH**	1805	3081
**Non-PSH**	3206	4894

**Table 2:** Estimate values of both PSH and DSH groups for AOB rate [3,21,22,23,24,25,26,27,28].

Author	PSH			DSH		
	nTX	mTX	sTX	nCT	mCT	sCT
G.M. Lopes Freire et al. 2016	272	−0.141	0.327	272	2.11	0.61
R.V de Sousa et al. 2014	732	2.441	0.255	732	0.387	0.274
S.R.F Hebling et al. 2008	659	3.492	0.234	513	1.035	0.305
Peres et al. 2007	359	2.154	0.26	359	0.744	0.377
C. Pimenta et al. 2023	87	0.085	0.554	87	1.69	0.706
A.C. Cardoso et al. 2014	839	3.538	0.229	778	2.239	0.323
E. Traebert et al. 2020	654	0.648	0.232	652	0.954	0.318
A. Germa et al. 2016	224	4.039	0.615	216	2.378	0.624
S. Davidopoulou et al. 2022	1185	1.725	0.421	1198	1.311	0.336

n = number of participants; m = mean; s = SD; TX = test PSH group; CT = DSH group.

**Table 3:** Results of meta-analysis of mean differences in log OR by type of sucking habit for AOB rate: weighted mean difference (WMD), standard error (SE), 95% confidence interval, z test (*p*-value), I^2^ index, Cochran’s Q statistic (*p*-value) for heterogeneity, and Egger’s test (*p*-value) for publication bias.

WMD	SE	95% CI	z (*p*-Value)	I^2^	Q_H_ (*p*-Value)	Egger (*p*-Value)
0.57	0.40	−0.21 1.35	0.150	99.9%	<0.001 ***	0.033 *

* *p* < 0.05; *** *p* < 0.001.

**Table 4:** Estimate values of both PSH and DSH groups for PCB rate [3,21,22,23,24,25,26,28,29,30].

Author	PSH			DSH		
	nTX	mTX	sTX	nCT	mCT	sCT
G.M. Lopes Freire et al. 2016	274	1.192	0.6231	274	−1.486	1.4500
R.V de Sousa et al. 2014	730	1.292	0.2600	732	0.143	0.3606
S.R.F Hebling et al. 2008	661	0.852	0.2098	514	−0.573	0.4880
R.R dos Santos et al. 2012	1298	1.07	0.1634	1304	0.427	0.1887
Peres et al. 2007	359	2.187	0.6120	359	−0.214	0.5071
C.Pimenta et al. 2023	87	0.45	0.8158	87	0.945	0.7631
M.C.B Macena et al. 2009	2503	0.381	0.1311	1800	0.020	0.2360
E.Traebert et al. 2020	654	0.965	0.2585	652	0.318	0.3843
A. Germa et al. 2016	224	1.751	0.3962	216	0.917	0.4199
S. Davidopoulou et al. 2022	1185	0.752	1.0499	1198	0.603	0.7710

n = number of participants; m = mean; s = SD; TX = test PSH group; CT = DSH group.

**Table 5:** Results of meta-analysis of mean differences in log OR by type of sucking habit for PCB rate: weighted mean difference (WMD), standard error (SE), 95% confidence interval, z test (*p*-value), I^2^ index, Cochran’s Q statistic (*p*-value) for heterogeneity, and Egger’s test (*p*-value) for publication bias.

WMD	SE	95% CI	z (*p*-Value)	I^2^	Q_H_ (*p*-Value)	Egger (*p*-Value)
0.98	0.13	0.72 1.24	<0.001 ***	99.9%	<0.001 ***	0.716

*** *p* < 0.001.

## Data Availability

The original contributions presented in this study are included in the article/Supplementary Material. Further inquiries can be directed to the corresponding author.

## Supplementary Materials

The following supporting information can be downloaded at: https://www.mdpi.com/article/10.3390/dj14010055/s1, Table S1: PRISMA Statement for Included studies; Table S2: Newcastle-Ottawa Scale (adapted for cross-sectional studies) for assessing the quality of non-randomised studies; Table S3: Newcastle-Ottawa Scale (adapted for cohort studies) for assessing the quality of non-randomised studies.
