# Supplementary material for: The Role of Digit- and Pacifier-Sucking Habits on Malocclusion Development in Children: Anterior Open Bite and Posterior Crossbite—A Systematic Review & Meta-Analysis
Source: Dent J (Basel). 2026 Jan 14;14(1):55. doi: 10.3390/dj14010055 (PMC12839743; doi:10.3390/dj14010055)
Supplement: Supplementary file 1 [file dentistry-14-00055-s001.zip › dentistry-4006181-supplementary.pdf]

Prisma Statement and Newcastle-Ottawa Scales:

	Checklist item	Articles											
		(G.M. Lopes Freire et al., 2016)	(de Sousa et al., 2014)	(Sandra Regina Facciolli Hebling et al., 2008)	(Renata Reis dos Santosa et al., 2012)	(Glazer et al., 2007)	(Pimenta et al., 2023)	(Carvalho Cardoso et al., 2014)	(MacEna et al., 2009)	(Tanny et al., 2021)	(Traebert et al., 2020)	(Germa et al., 2016)	(Davidopoulou et al., 2022)
**TITLE**													
Title	Identify the report as a systematic review	NO	NO	NO	NO	NO	NO	NO	NO	NO	NO	NO	NO
**ABSTRACT**													
Abstract	See the PRISMA 2020 for Abstract checklist	YES	YES	YES	YES	YES	YES	YES	YES	YES	YES	YES	YES
**INTRODUCTION**													
Rationale	Describe the rationale for the review in the context of existing knowledge.	YES (p. 301-302)	YES (p.336)	YES (p. 75)	YES (p. 311-312)	YES (p. 344)	YES (p.28 - 29)	YES (p. 219-220)	YES (p. 357)	YES (p. 1-2)	YES (p. 1-2)	YES (p. 832-833)	YES (p.2)
Objectives	Provide an explicit statement of the objective(s) or question(s) the review addresses.	YES (p. 302)	YES (p.336)	YES (p. 75)	YES (p. 312)	YES (p.344-345)	YES (p. 29)	YES (p. 220)	YES (p. 357)	YES (p. 2)	YES (p. 2)	YES (p.833)	YES (p.2)
**METHODS**													
Eligibility criteria	Specify the inclusion and exclusion criteria for the review and how studies were grouped for the syntheses.	YES (p. 302)	YES (p.337)	YES (p. 76)	YES (p. 312)	YES (p.345)	YES (p. 29)	YES (p. 220-221)	YES (p. 358)	YES (p. 2)	YES (p. 3)	YES (p. 833-834)	YES (p.2)

Information sources	Specify all databases, registers, websites, organisations, reference lists and other sources searched or consulted to identify studies. Specify the date when each source was last searched or consulted.	YES (p. 302 – 303)	YES (p.337-338)	YES (p. 76)	YES (p. 312-313)	YES (p.344-345)	YES (p. 29)	YES (p. 220-221)	YES (p. 358)	YES (p. 2-3)	YES (p. 3-4)	YES (p. 834)	YES (p.2-3)
Search strategy	Present the full search strategies for all databases, registers and websites, including any filters and limits used.	NO	NO	NO	NO	NO	NO	NO	NO	NO	NO	NO	NO
Selection process	Specify the methods used to decide whether a study met the inclusion criteria of the review, including how many reviewers screened each record and each report retrieved, whether they	YES (p. 302)	YES (p.337)	YES (p. 76)	YES (p. 312)	YES (p.345-346)	YES (p. 29-30)	YES (p. 221)	YES (p. 358)	YES (p. 2)	YES (p. 3-4)	YES (p. 834)	YES (p.3)

	worked independently, and if applicable, details of automation tools used in the process.												
Data collection process	Specify the methods used to collect data from reports, including how many reviewers collected data from each report, whether they worked independently, any processes for obtaining or confirming data from study investigators, and if applicable, details of automation tools used in the process.	YES (p. 302 - 303)	YES (p.337-338)	YES (p. 76)	YES (p. 312-313)	YES (p.345-346)	YES (p. 29-30)	YES (p. 221-222)	YES (p. 358)	YES (p. 2-3)	YES (p. 3-4)	YES (p. 834)	YES (p.3)
Data items	List and define all outcomes for which data were sought. Specify whether all results that were compatible with each outcome domain. In each study were	YES (p. 302 - 303)	YES (p.338)	YES (p. 76)	YES (p. 312-313)	YES (p.346)	YES (p. 29-30)	YES (p. 221-222)	YES (p. 358)	YES (p. 2-3)	YES (p. 3-4)	YES (p. 834)	YES (p.3)

	sought (e.g. for all measures, time points, analyses), and if not, the methods used to decide which results to collect.												
	List and define all other variables for which data were sought (e.g. participant and intervention characteristics, funding sources). Describe any assumptions made about any missing or unclear information.	YES (p. 302 - 303)	YES (p.338)	YES (p. 76)	YES (p. 312-313)	YES (p.346)	YES (p. 29-30)	YES (p. 221-222)	YES (p. 358)	YES (p. 2-3)	YES (p. 3-4)	YES (p. 834)	YES (p.3)
Study risk of bias assessment	Specify the methods used to assess risk of bias in the included studies, including details of the tool(s) used, how many reviewers assessed each study and whether they worked independently, and if applicable, details of automation tools	NO	NO	YES (p. 76)	YES (p. 313)	YES (p.346)	YES (p. 30)	YES (p. 221)	YES (p. 358)	YES (p. 2-3)	YES (p. 4)	YES (p. 834)	YES (p.3)

	used in the process.												
Effect measures	Specify for each outcome the effect measure(s) (e.g. risk ratio, mean difference) used in the synthesis or presentation of results.	YES (p. 303-304)	YES (p.338-339)	YES (p. 77-78)	YES (p. 313)	YES (p.346)	YES (p. 30)	YES (p. 222-224)	YES (p. 359)	YES (p. 3)	YES (p. 5-6)	YES (p. 835-836)	YES (p.3-4)
Synthesis methods	Describe the processes used to decide which studies were eligible for each synthesis (e.g. tabulating the study intervention characteristics and comparing against the planned groups for each synthesis (item #5)).	YES (p. 302)	YES (p. 337)	YES (p. 76)	YES (p. 312)	YES (p.345-346)	YES (p. 29)	YES (p. 221)	YES (p. 358)	YES (p. 2-3)	YES (p. 3)	YES (p. 834)	YES (p.2)
	Describe any methods required to prepare the data for presentation or synthesis, such as handling of missing summary statistics, or data conversions.	YES (p. 303)	NO	NO	NO	NO	YES (p.30)	NO	NO	YES (p. 3)	YES (p. 4)	YES (p. 834)	YES (p.3-4)

	Describe any methods used to tabulate or visually display results of individual studies and syntheses.	YES (p. 303-304)	YES (p.338-340)	YES (p.77-78)	YES (p. 314-316)	YES (p.346-348)	YES (p. 30-31)	YES (p. 223-224)	YES (p. 359)	YES (p. 3-4)	YES (p. 5-6)	YES (p. 835-836)	YES (p.3-7)
	Describe any methods used to synthesize results and provide a rationale for the choice(s). If meta-analysis was performed, describe the model(s), method(s) to identify the presence and extent of statistical heterogeneity, and software package(s) used.	YES (p. 303)	YES (p.338)	YES (p. 77-78)	YES (p. 313)	YES (p.347)	YES (p. 30)	YES (p. 223-224)	YES (p. 359)	YES (p. 3-4)	YES (p. 6)	YES (p. 834)	YES (p.3-7)
	Describe any methods used to explore possible causes of heterogeneity among study results (e.g. subgroup analysis, meta- regression).	NO	NO	NO	NO	NO	NO	NO	NO	YES (p.3)	NO	NO	NO
	Describe any sensitivity analyses conducted to assess	NO	NO	NO	NO	NO	NO	NO	NO	NO	NO	NO	NO

	robustness of the synthesized results.												
Reporting bias assessment	Describe any methods used to assess risk of bias due to missing results in a synthesis (arising from reporting biases).	NO	NO	NO	NO	NO	NO	NO	NO	NO	NO	NO	NO
Certainty assessment	Describe any methods used to assess certainty (or confidence) in the body of evidence for an outcome.	NO	NO	NO	NO	NO	NO	NO	NO	YES (p. 3-4)	NO	NO	NO
**RESULTS**													
Study selection	Describe the results of the search and selection process, from the number of records identified in the search to the number of studies included in the review, ideally using a flow diagram.	NO	NO	NO	NO	NO	NO	NO	NO	YES (p.3)	NO	YES (p. 834)	NO
	Cite studies that might appear to meet the inclusion criteria, but which were excluded and explain	NO	YES (p. 338)	YES (p. 76)	NO	NO	NO	NO	NO	YES (p.3)	NO	YES (p.834)	NO

	why they were excluded.												
Study characteristics	Cite each included study and present its characteristics.	YES (p. 303-304)	YES (p. 338 – 339)	YES (p. 76-77)	YES (p. 314-316)	YES (p.346)	YES (p. 30)	YES (p. 222-224)	YES (p. 359)	YES (p. 3-4)	YES (p. 5)	YES (p. 835-836)	YES
Risk of bias in studies	Present assessments of risk of bias for each included study.	NO	NO	NO	NO	NO	NO	NO	NO	NO	NO	NO	NO
Results of individual studies	For all outcomes, present, for each study: (a) summary statistics for each group (where appropriate) and (b) an effect estimate and its precision (e.g. confidence/credible interval), ideally using structured tables or plots.	YES (p. 303-304)	YES (p. 338 – 340)	YES (p. 77-78)	YES (p. 314-316)	YES	YES (p. 30-31)	YES	YES	YES	YES	YES (p. 835-836)	YES (p.3-7)
Results of syntheses	For each synthesis, briefly summarise the characteristics and risk of bias among contributing studies.	NO	NO	NO	NO	NO	NO	NO	NO	NO	NO	NO	NO
	Present results of all statistical syntheses conducted. If meta-	NO	NO	NO	YES (p.314-316)	YES	YES	YES (p.223)	YES	YES	YES	NO	NO

	analysis was done, present for each the summary estimate and its precision (e.g. confidence/credible interval) and measures of statistical heterogeneity. If comparing groups, describe the direction of the effect.												
	Present results of all investigations of possible causes of heterogeneity among study results.	NO	NO	NO	NO	NO	NO	NO	NO	YES	NO	NO	NO
	Present results of all sensitivity analyses conducted to assess the robustness of the synthesized results.	NO	NO	NO	NO	NO	NO	NO	NO	NO	NO	NO	NO
Reporting biases	Present assessments of risk of bias due to missing results (arising from reporting biases) for each synthesis assessed.	NO	NO	NO	NO	NO	NO	NO	NO	NO	NO	NO	NO

Certainty of evidence	Present assessments of certainty (or confidence) in the body of evidence for each outcome assessed.	NO	NO	NO	NO	NO	NO	NO	NO	NO	NO	NO	NO
**DISCUSSION**													
Certainty of evidence	Provide a general interpretation of the results in the context of other evidence.	YES (p.304)	YES (p.339-340)	YES (p. 78-79)	YES (p. 316)	YES (p.348-349)	YES (p. 31-32)	YES (p.224)	YES (p.360)	YES (p.4)	YES (p.6-7)	YES (p. 836-838)	YES (p.7-9)
	Discuss any limitations of the evidence included in the review.	YES (p.304)	YES (p.339-340)	YES (p. 78-79)	YES (p. 316)	YES (p.348-349)	YES (p. 32)	YES (p.224)	YES (p.360)	YES (p.4)	YES (p.6-7)	YES (p. 837)	YES (p.8-9)
	Discuss any limitations of the review processes used.	NO	NO	NO	NO	YES	NO	NO	NO	NO	NO	NO	NO
	Discuss implications of the results for practice, policy, and future research.	YES (p.304)	YES (p.339-340)	YES (p. 78-79)	YES (p. 316-317)	YES (p. 349)	YES (p. 32)	YES (p.224)	YES (p.360)	YES (p.4-5)	YES (p.6-7)	YES (p. 837-838)	YES (p.9)
**OTHER INFORMATION**													
Registration and protocol	Provide registration information for the review, including register name and registration number, or state	NO	NO	NO	NO	NO	NO	NO	NO	NO	NO	NO	NO

	that the review was not registered.												
	Indicate where the review protocol can be accessed, or state that a protocol was not prepared.	NO	NO	NO	NO	NO	NO	NO	NO	NO	NO	NO	NO
	Describe and explain any amendments to information provided at registration or in the protocol.	NO	NO	NO	NO	NO	NO	NO	NO	NO	NO	NO	NO
Support	Describe sources of financial or non-financial support for the review, and the role of the funders or sponsors in the review.	NO	NO	NO	YES	NO	NO	NO	NO	NO	NO	YES	YES
Competing interests	Declare any competing interests of review authors.	NO	NO	NO	NO	NO	NO	NO	NO	NO	NO	NO	NO
Availability of data, code and other materials	Report which of the following are publicly available and where they can be found template data collection forms; data extracted from included studies;	NO	NO	NO	NO	NO	NO	NO	NO	NO	NO	NO	NO

	data used for all analyses; analytic code; any other materials used in the review.												
**TOTAL**		19/42	19/42	20/42	21/42	20/42	21/42	19/42	20/42	26/42	21/42	23/42	21/42

Table S1: PRISMA Statement for Included studies

Table S2: Newcastle-Ottawa Scale (adapted for cross-sectional studies) for assessing the quality of non-randomised studies.[21,23,25,30,3,22,27,31,29,26,28]

Newcastle-Ottawa Scale (Adapted for cross-sectional studies)

This scale is used as a method to evaluate the quality of non-randomized studies such as case-control, cohort and cross-sectional studies (with an adapted scoring system). The results are shown in Table S2 below, where a row is filled in green for "good quality" and in amber for "fair quality" studies (Newcastle-Ottawa Quality Assessment Form for Cohort Studies [Internet]).

Newcastle-Ottawa Assessment Scale (Adapted for cross-sectional studies)								
Study	Selection (Max 5 stars)				Comparability (Max 2 stars)	Outcome (Max 3 stars)		Total
	Representativeness of the sample	Sample Size	Non-respondents	Ascertainment of the exposure		Assessment of outcome	Statistical test	
G.M. Lopes Freire et al. 2016	★	★		★	★	★	★	6
R.V de Sousa et al. 2014	★	★	★	★	★ ★	★	★	8
S.R.F Hebling et al. 2008	★	★	★	★	★ ★	★	★	8
R.R dos Santos et al. 2012	★	★	★	★	★	★	★	7
Peres et al. 2007	★	★	★	★	★★	★	★	8
C. Pimenta et al. 2023		★	★	★	★	★	★	6
A.C Cardoso et al. 2014	★	★	★	★	★★	★	★	8
M.C.B Macena et al. 2009	★	★		★	★	★	★	6
L. Tanny et al. 2020	★	★	★	★	★★	★	★	8
E. Traebert et al. 2020	★	★	★	★	★★	★	★	8
S. Davidopoulou et al. 2022	★	★	★	★	★★	★	★	8

Table S3: Newcastle-Ottawa Scale (adapted for cohort studies) for assessing the quality of non-randomised studies.[24]

Newcastle-Ottawa Assessment Scale									
Study	Selection (Max 4 stars)				Comparability (Max 2 stars)	Outcome (Max 3 stars)			Total
	Representativeness of exposed cohort	Selection of non-exposed cohort	Ascertainment of exposure	Outcome of interest not present at start of study		Assessment of outcome	Adequate follow-up duration	Adequate completeness of follow-up	
A.Germa et al. 2016	★	★	★	★	★★	★	★	★	9
